# Frataxin‐deficient neurons and mice models of Friedreich ataxia are improved by TAT‐MTScs‐FXN treatment

**DOI:** 10.1111/jcmm.13365

**Published:** 2017-10-05

**Authors:** Elena Britti, Fabien Delaspre, Anat Feldman, Melissa Osborne, Hagar Greif, Jordi Tamarit, Joaquim Ros

**Affiliations:** ^1^ Departament de Ciències Mèdiques Bàsiques IRBLleida Universitat de Lleida Lleida Spain; ^2^ BioBlast‐Pharma Ltd. TelAviv Israel; ^3^ The Jackson Laboratory Bar Harbor Maine USA

**Keywords:** frataxin, dorsal root ganglia neurons, α‐fodrin/heat‐shock protein 60, succinate dehydrogenase, friedreich ataxia

## Abstract

Friedreich ataxia (FA) is a rare disease caused by deficiency of frataxin, a mitochondrial protein. As there is no cure available for this disease, many strategies have been developed to reduce the deleterious effects of such deficiency. One of these approaches is based on delivering frataxin to the tissues by coupling the protein to trans‐activator of transcription (TAT) peptides, which enables cell membranes crossing. In this study, we tested the efficiency of TAT‐MTScs‐FXN fusion protein to decrease neurodegeneration markers on frataxin‐depleted neurons obtained from dorsal root ganglia (DRG), one of the most affected tissues. In mice models of the disease, we tested the ability of TAT‐MTScs‐FXN to penetrate the mitochondria and its effect on lifespan. In DRG neurons, treatment with TAT‐MTScs‐FXN increased cell survival, decreased neurite degeneration and reduced apoptotic markers, such as α‐fodrin cleavage and caspase 9 activation. Also, we show that heat‐shock protein 60 (HSP60), a molecular chaperone targeted to mitochondria, suffered an impaired processing in frataxin‐deficient neurons that was relieved by TAT‐MTScs‐FXN addition. In mice models of the disease, administration of TAT‐MTScs‐FXN was able to reach muscle mitochondria, restore the activity of the succinate dehydrogenase and produce a significant lifespan increase. These results support the use of TAT‐MTScs‐FXN as a treatment for Friedreich ataxia.

## Introduction

Friedreich ataxia (FA) is the most common autosomal recessive ataxia known to date, with a population frequency of 1–2/50,000 1 and early life mortality caused mainly by cardiomyopathy 2. FA is attributed to partial depletion of the mitochondrial protein Frataxin, caused by a trinucleotide GAA expansion in intron 1 of the Frataxin gene. When both Frataxin alleles contain the intron 1 expansion, frataxin level is reduced below the normal threshold and FA is overt 3. Additionally, increased number of GAA repeats (100–1700 in FA patients) is associated with more severe clinical outcome 2. Frataxin is a nuclear encoded protein that is translocated to the mitochondria due to a mitochondrial targeting sequence (MTS) that is expressed on its N‐terminus 4, 5. Frataxin biological function is not completely elucidated; however, it was found to regulate the biogenesis of mitochondrial Fe‐S clusters 6, 7 Fe‐S clusters serve as prosthetic groups for a series of enzymes including, among others, energy metabolism enzymes such as aconitase 8 or proteins of the respiratory chain including succinate dehydrogenase 9. These enzymes were found to be impaired in FA patients 10. Nevertheless, frataxin may have additional roles as Fe‐S deficiency is not a universal consequence of frataxin deficiency 11, 12.

Leading drug candidates for FA are now in phase 2 clinical trials, but currently, no cure is available. Drug candidates include, among others, mitochondrial function enhancers, antioxidant and neuroprotection agents, enhancers of frataxin gene expression and frataxin replacement. The latter consists in delivering frataxin to cells using the HIV‐trans‐activator of transcription (TAT) as carrier molecule 13. Using a conditional‐KO mouse model in which Frataxin was depleted in the brain 14, intraperitoneal (IP) TAT‐FXN injections showed evidence of mitochondrial localization and rescued the fatal phenotype. TAT‐FXN has also been shown to cross the blood–brain barrier and protect dopaminergic neurons in a mouse Parkinson disease model after IP injections 15.

Most of the mitochondrial proteins are synthesized with mitochondrial targeting sequence (MTS), a positively charged amphipathic α‐helix structure 16 typically 15–30 amino acid long, which allows their import from the cytoplasm into the mitochondria through the translocase of the outer and inner mitochondrial membranes (TOM/TIM). Upon translocation through TOM complex, the inner mitochondrial membrane potential (Δψ_m_, negative on matrix side) 17 and the ATP‐dependent action of mitochondrial heat‐shock protein 70 (mtHSP70) drive the protein import into matrix space 18 where MTS is recognized by mitochondrial processing proteases (MPP), retaining the mature protein within the mitochondria. In this context, one of the mitochondrial proteins imported into the mitochondrial matrix is HSP60, a molecular chaperone which is highly conserved from bacteria to humans. When the importing machinery is altered, HSP60 precursor form (MTS‐HSP60) accumulates and can be detected by Western blot 19, 20. Frataxin precursor ‐FXN_1‐210_‐ contains an unusual 80‐amino acid‐long MTS with several cleavage sites, which undergoes two‐step processing upon entry into the mitochondria, leading to at least a transient processing intermediate ‐FXN_42–210_‐ before of an alternative processing ‐FXN_56–210_‐ 21 and/or the mature form ‐FXN_81–210_‐ 22. Recently, a construct was designed (TAT‐MTScs‐FXN) consisting in human Frataxin mature protein ‐FXN_81–210_‐ sequence coupled with TAT sequence, that promotes cellular internalization and the mitochondrial targeting sequence of citrate synthase (MTScs), allowing efficient mitochondrial cleavage. This fusion protein was used to deliver frataxin into mitochondria of human Bjab lymphoma cell line as well as into cell cultures obtained from FA patients 23. The results showed that frataxin was delivered with higher efficiency compared to TAT‐MTScs‐FXN, a construction carrying the native MTS of human frataxin.

In this study, we have used our model of frataxin‐deficient DRG neurons 24 to show that TAT‐MTScs‐FXN is able to reach mitochondria of DRG neurons, partially rescuing cell viability, reducing neurite degeneration and decreasing α‐fodrin cleavage, a marker of apoptotic process. These neurodegenerative markers were observed in our model as a consequence of altered calcium homoeostasis 24. Moreover, when TAT‐MTScs‐FXN was injected in two mice models of FA 14, 25, with either 4 or 10 mg/kg, functional frataxin was observed in muscle tissue, where it was able to recover the activity of succinate dehydrogenase (SDH) to the levels of heterozygous healthy mice. Reduced SDH activity impairs mitochondrial function and has been demonstrated to occur in Friedreich ataxia heart autopsies 10. TAT‐MTScs‐FXN is also able to promote an increase in the mean survival of mice treated with 10 mg/kg. The results obtained with both cell and animal models indicate that this approach can be a promising strategy for FA treatment.

## Materials and methods

### Plasmids and production of lentiviral particles

Reduction in frataxin in cultured DRG neurons was achieved by short‐hairpin RNA‐interfering sequences. Lentiviral vectors are routinely produced as described in our previous publication 24. The shRNA lentiviral plasmids (pLKO.1‐puro) for human/mouse/rat frataxin were purchased from Sigma‐Aldrich (Merck. Madrid. Spain). The RefSeq used was NM_008044, which corresponds to mouse frataxin. The clones used were TRCN0000197534, TRCN0000006137 and referred in this paper as Fxn1 and Fxn2, respectively). A non‐targeted scrambled sequence (the vector SHC002) served as a control (here referred as Scr).

### Isolation and culture of DRG sensory neurons

DRGs were extracted and purified as previously described in Mincheva‐Tasheva *et al*., 2014 24. Briefly, DRGs were extracted from neonatal rats (P3–P4) and dissociated with 0.05% trypsin (Sigma‐Aldrich). Ganglia were mechanically disrupted with a pipette tip until obtaining a single cell suspension in culture media supplemented with DNAse I grade II at final concentration 3 mg/ml (Roche Diagnostics, Madrid, Spain), which was centrifuged at 800×g r.p.m. (302 rcf) through 7,5% BSA solution (Sigma‐Aldrich) for 5 min., followed by resuspension in enriched neurobasal culture media (Thermo Fisher Scientific—TFS, Madrid, Spain) (NBMc) consisting of 2% horse serum (TFS), 2% B27 supplement (TFS), 0.5 mM L‐glutamine (TFS), 100 U/ml penicillin plus 100 ng/ml streptomycin (TFS) and supplemented with murine β‐nerve growth factor at 50 ng/ml (PeproTech, New Jersey, USA). To prevent growth of non‐neuronal cells, culture media were supplemented with the anti‐mitotic agent Aphidicolin (Sigma‐Aldrich) at final concentration 3,4 μg/ml. After 1 hr of pre‐plating in a p60 tissue dish (Corning Inc., Fisher Scientific. Madrid. Spain) at 37°C/5%CO2, the cells were then plated in a 24‐well tissue dish (Corning Incorporated) pre‐treated with 0,1 mg/ml of collagen (Sigma‐Aldrich) at a cell density of 30,000 cells/well. After 1–2 days, lentiviral transduction was performed, and lentivirus particles (20 ng/1000 cells) were added. After transduction, allowed to proceed for 6 hrs, media containing lentivirus were substituted by fresh culture media. TAT‐MTScs‐frataxin was added to cultures at 12 hrs or 48 hrs after ending lentivirus transduction protocol and used at the concentrations indicated in each figure. Usually, concentrations of 1, 3 or 7 μg/ml were used. The culture medium was not changed after the addition of TAT‐MTScs‐frataxin.

### Immunofluorescence staining

#### Immunocytochemistry of DRG neurons

Cell cultures were fixed in 4% paraformaldehyde and incubated overnight at 4°C with specific antibodies at indicated dilutions: rabbit anti‐frataxin (1:50) from Santa Cruz Biotechnology (Dallas, USA) and mouse anti‐OxPhos (1:50) from Novex (Invitrogen, Madrid, Spain)). Anti‐rabbit or antimouse secondary antibodies conjugated with Alexa Fluor 555 or Alexa Fluor 488 (Invitrogen, Madrid, Spain), respectively, were used at 1:300 dilutions and incubated for 2 hrs at room temperature, protected from light. For cell nucleus staining, the fixed cells were incubated for 5 min. with DAPI. Micrographs were taken using an Olympus FluoView 500 (60×).

#### Immnunohistochemistry of heart tissue

Hearts were obtained from wild‐type mice (C57BL/6J) and MCK mice treated with vehicle or 10 mg/Kg TAT‐MTScs‐FXN. Unstained slides were deparaffinized and rehydrated. Heat‐induced epitope retrieval was performed with Diva antigen retrieval buffer (Biocare Medical Pacheco, CA, USA). Following a rinse, a protein blocking step was performed with Sniper protein block (Biocare Medical). Slides were again rinsed and anti‐frataxin antibody, ab175402 from Abcam (Cambridge, UK), was applied at 1:50 dilution for 60 min. Slides were rinsed. Endogenous peroxidase was blocked using Peroxidase 1 [Biocare Medical]. After a rinse, the secondary antibody, biotinylated goat anti‐rabbit IgG (1:500 dilution) from Jackson Immunoresearch (ref 111‐065‐144) was applied for 30 min. The slides were rinsed, and ABC Elite was added for 30 min., followed by another rinse. Colour development was achieved by adding Sigma Fast DAB solution for 5 min. Slides were rinsed, counterstained with haematoxylin and coverslipped. All rinse steps were performed with TBS‐Autowash (Biocare Medical).

### Survival and neurite degeneration analyses

Neuronal survival was measured with a ×16 lens and cross‐marked wells as described 24. Neurons at zero and 5 days were counted at four fields per cross‐marked well, using three wells per condition tested. Experiments were repeated at least three times. Neurite degeneration was measured with a ×32 lens and a grid, which was created over each image with NIH Image J with the grid plugin (Image size 680 × 512 and line area 10,000 pixels2). Healthy and degenerated neurites (displaying neurofilament aggregates) were counted at three grid fields per image and three images per well were analysed. For each condition, we used three different wells. Experiments were repeated at least three times.

### Animal care

All experiments were conducted in accordance with the protocols described by the National Institutes of Health's Guide for the Care and Use of Animals and were approved by JAX institutional animal care and use committees. Mice were ear notched for identification and housed in individually and positively ventilated polycarbonate cages with HEPA‐filtered air at a density no greater than of four to five mice per cage. Pine shaving‐based corn cob bedding was used, and cages were changed every 2 weeks. The animal room was lighted entirely with artificial fluorescent lighting, with a controlled 12‐hrs light/dark cycle (7 a.m. to 7 p.m. light). The normal temperature and relative humidity ranges in the animal rooms were 22 ± 4°C and 50 ± 15%, respectively. The animal rooms were set to have 15 air exchanges per hour. Filtered tap water, acidified to a pH of 2.8–3.2 and LabDiet 5LL4 were provided *ad libitum*.

### Animals breeding and genotyping

*FA‐KO mouse model* (B6.Cg‐Tg. (Fxn)1 Sars FXN tm1mkn/J, Jax stock #18299): Mice are homozygous to the mouse Frataxin gene knockout and hemizygous to the human FA‐related Frataxin gene, containing 500 GAA trinucleotide repeats. Mice, licensed and routinely bred at the Jackson laboratory, were produced by crossing heterozygous Frataxin KO mice with mice heterozygous for the Frataxin KO and hemizygous human FA‐related Frataxin gene. This model is characterized by a low human Frataxin expression level, showing normal behavioural, biochemical or histological measures 25.

*FA‐Conditional‐KO mouse model* (Mutant Mck‐Cre‐Fxn^L3/L−^, Jax Custom Breeding stock 371001): Mice, licensed to Bioblast Pharma, carrying the floxed Frataxin allele, were generated at the laboratory of Koeing and Puccio as described 14. Sperm from two male mice were used for IVF with inbred C57BL/6J (JAX Stock No: 000664) as oocyte donors, performed with The Jackson Laboratory common procedures. Two Jax stocks (Stock 006475 B6.FVB(129S4)‐Tg(Ckmm‐cre)5Khn/J and Stock 16842 B6.Cg‐Fxntm1Mkn/J) were used for breeding as described below.

Mutant animals (compound heterozygotes at the murine Frataxin locus harbouring the floxed allele Fxntm2Mkn and the null allele Fxntm1Mkn and carrying the Tg (Ckmm‐cre) 5Khn) were generated by intercrossing animals homozygous for the floxed allele Fxntm2Mkn with animals heterozygous for the null allele Fxntm1Mkn and hemizygous for the Tg (Ckmm‐cre) 5Khn transgene. Mutants were identified by PCR genotyping of genomic DNA isolated from tail biopsies. To genotype the Fxntm1Mkn targeted mutation, multiplexed PCR was performed on DNA isolated from tail biopsies using the following primers: Common‐F: 5′‐CTG TTT ACC ATG GCT GAG ATC TC‐3′; WT‐R: 5′‐CCA AGG ATA TAA CAG ACA CCA TT‐3′; MUT‐R: 5′‐CGC CTC CCC TAC CCG GTA GAA TTC‐3′ generating at 245‐bp mutant product and 499‐bp wild‐type product. To identify animals harbouring the floxed allele, the following primer sets were used: FxnflF: 5′‐GGTCCATATAGTGCGTTCCAG‐3′ and FxnflR: 5′‐CGCTCTCTTCAGAC AGACCA‐3′ generating a 300‐bp mutant product and a 256‐bp wild‐type product. Animals harbouring the Tg(Ckmm‐cre) 5Khn transgene were identified with the primer set: Ckmm‐F: 5′‐GTGAAACAGCATTGCTGTCACTT‐3′ and Ckmm‐R: 5′‐TAAGTCTGAACCCGGTCTGC‐3′ generating a 450‐bp mutant product. According to a recent paper 26 (Perdomini, 2014), mutant Mck‐Cre‐Fxn^L3/L−^ mice are asymptomatic at wean and exhibit weight loss and die of progressive cardiac failure at 65 ± 10 days of age.

### Administration of TAT‐MTScs‐frataxin to mice and survival monitoring

Treatment was initiated at time of weaning (at 3–4 weeks of age). Mice were intravenously (IV) injected twice weekly (at least 3 days apart) with either vehicle (containing 50 mM Tris‐HCl, 300 mM NaCl, 10% glycerol pH 8), or with TAT‐MTScs‐FXN at doses of 4 mg/kg or 10 mg/kg.

For survival experiments, treatment was initiated at time of weaning (3–4 weeks of age). MCK mice were intraperitoneally (IP) injected twice weekly following the protocol described above. MCK mice survival was monitored daily. Additionally, mice that lost >15% weekly weight were killed and registered as death incidents. Significance was defined using log‐rank (Mantel–Cox) test.

### Isolation of mitochondria from muscle tissue

Mice tissue was extracted, and the mitochondrial fraction was isolated using mitochondria isolation kit (Abcam, ab110168) according to the manufacturer's protocol. Mitochondrial pellets were preserved at −80°C.

### Measurement of frataxin amounts in muscle mitochondrial fractions

Human frataxin level was analysed in isolated mitochondrial fractions using Abcam ab176112 ELISA kit, according to the manufacturer's instructions.

### Mitochondrial complex II activity assay

Complex II activity (succinate dehydrogenase, or SDH) in mitochondrial fractions was determined using a commercially available kit (BioVision, K660‐100, Milpitas, California, USA) according to manufacturer's instructions. Mitochondrial pellets were resuspended with 90–150 μl succinate dehydrogenase (SDH) buffer, according to mitochondrial pellet amount (0.2V of resuspended volume) was used in duplicates. Protein was determined by the Bradford assay. Enzyme activity was followed at 600 nm and measured every 2 min. up to 20 min. Specific activity was given as ΔOD per mg of protein.

### Western blot analysis

Western blot analysis were performed using equal volumes of mitochondrial extracts, diluted in sample buffer, separated using 4–12% gradient SDS‐PAGE gels. External human Frataxin protein was used as a positive control (5 μg/well). COX‐IV mitochondrial marker was detected using specific antibody from R&D Systems Abingdon, UK (MAB6980) diluted 1:1000 in blocking buffer at room temperature for 1.5 hrs. Following analysis, nitrocellulose membranes were washed, striped and subjected to Frataxin detection, using Frataxin antibody from Abnova, Walnut, CA, USA (H00002395‐M02). Both Frataxin and COX‐IV signals were quantified using Image J software, and Frataxin signal was normalized to COX‐IV signal. For DRGs, cells were rinsed three times in ice‐cold PBS (pH 7.4) and lysed with 2% SDS, 125 mM Tris and protease inhibitors (Roche) to obtain crude extracts used for SDS–polyacrylamide gel electrophoresis. In Western blot analysis, PVDF membranes were used for HSP60, fodrin, procaspase 9 and actin detection and nitrocellulose membranes for Frataxin detection. Monoclonal antibody against HSP60 was from Acris‐Origene Technology Rockville, MD, USA (TA326374) used at 1/6000 dilution; α‐fodrin cleavage products antibody was from ENZO Life Sciences (Farmingdale, NY, USA) used at 1:1000 dilution, and anti‐caspase 9 monoclonal antibody was from Cell Signaling Technology (9508) used at 1:1000 dilution. Frataxin was detected using anti‐Frataxin antibody from Santa Cruz Biotechnology 1:250 dilution. Fodrin, HSP60, procaspase 9 and frataxin signals were normalized to actin signal, using anti‐β‐actin antibody from Chemicon‐EMD Millipore, Madrid, Spain at 1:50,000 dilution.

### Data analysis

The data obtained from at least three independent experiments were used for statistical analyses. Values were expressed as mean ±S.E.M. (error bars). One‐way anova was used to assess survival, neurite degeneration between groups. If the anova test was statistically significant, we performed post hoc pairwise comparisons using the Bonferroni test. The *P*‐values lower than 0.05(∗), 0.01(∗∗) or 0.001 (∗∗∗) were considered significant. For *in vivo* studies, statistical analyses were performed using Statistical Package for the Social Sciences (SPSS software version 22. licence number f11340ae08e3b46186e4).

## Results

### TAT‐MTScs‐FXN penetrates DRG neurons

**Figure 1:**
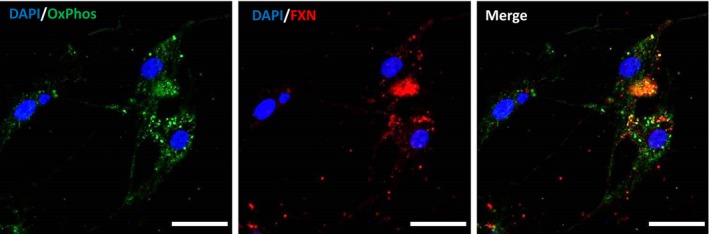
TAT‐MTScs‐FXN penetrates DRG neurons. Immunofluorescent staining for frataxin (red) and OxPhos (green) in DRG neurons (Fxn2) treated with 7 μg/ml TAT‐MTScs‐FXN 12 hrs after lentiviral transduction. Images were obtained after 5 days of culture. OxPhos staining was used to visualize mitochondria. Nuclei were stained with DAPI.

TAT‐fusion proteins have been used to successfully deliver proteins and peptides into cells as treatment for various diseases. Although the mechanism is unknown, transduction mechanism requires an initial ionic interaction between TAT peptide and the cell surface, followed by TAT‐fusion protein internalization through lipid‐raft‐dependent micropinocytosis 27. Using our model of rat primary culture, we tested the ability of the TAT‐MTScs‐FXN to penetrate frataxin‐deficient DRG neurons. DRG neurons were transduced with lentiviruses carrying either Fxn1 or Fxn2 shRNAs to reduce the endogenous expression level of frataxin or lentivirus carrying Scr shRNA to use it as control condition for frataxin level. Efficient reduction in endogenous levels of frataxin was achieved by Fxn1 and Fxn2 interference RNAs (shown in Fig. S1). After 48 hrs of lentiviral transduction, TAT‐MTScs‐FXN (7 μg/ml) or vehicle solution was added to cultures. To evaluate the presence and mitochondrial location of frataxin in these cells, cultures were co‐stained by immunofluorescence with anti‐frataxin and anti‐OxPhos antibodies. The latter being a cocktail of antibodies that recognize different subunits of the mitochondrial respiratory chain. Frataxin is almost undetectable in frataxin‐depleted DRG neurons (data not shown). In contrast, frataxin can be detected after TAT‐MTScs‐FXN treatment, indicating that the fusion protein is able to penetrate frataxin‐deficient cells (Fig. 1). Moreover, the punctate pattern of frataxin staining in the cell soma and along the neurites mostly co‐localize with mitochondrial marker, which shows that TAT‐MTScs‐FXN is targeted to the mitochondria.

### TAT‐MTScs‐FXN processing in DRG neurons

**Figure 2:**
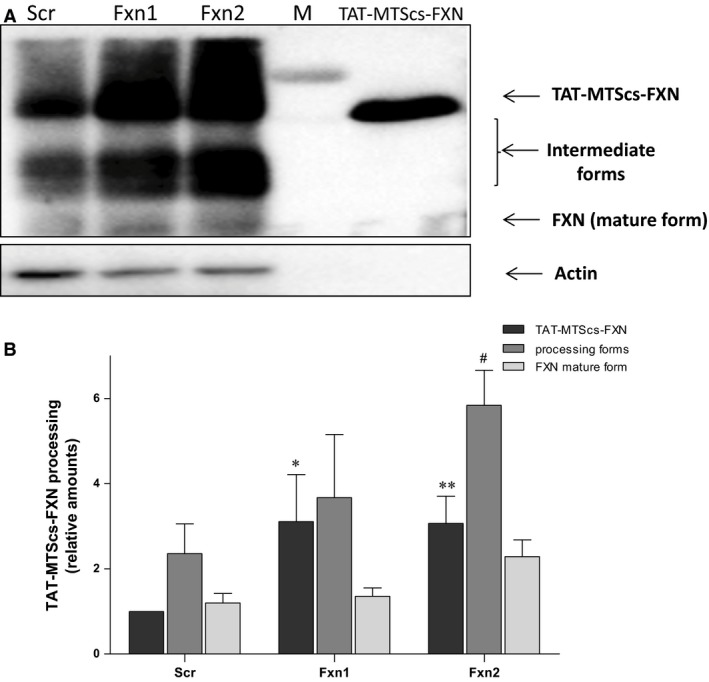
Western blotting of TAT‐MTScs‐FXN processing. (**A**) Cells from Scr, Fxn1 and Fxn2 treated with 7 μg/ml TAT‐MTScs‐FXN 12 hrs after lentiviral transduction were lysed after 5 days of culture and blotted to detect frataxin levels (β‐actin was used for normalization). Results indicate penetration and processing of TAT‐MTScs‐FXN in all cultures. Differences in intermediate forms are evident in Fxn1 and Fxn2 compared to control (Scr) conditions. The lane on the right is to show the molecular weight of the intact TAT‐MTScs‐frataxin form. Lane M indicates the position of the molecular weight marker of 25 kD. (**B**) The histogram illustrates the relative amounts of frataxin forms when TAT‐MTScs‐frataxin (7 μg/ml) was added to the cultures 12 hrs after lentivirus withdrawal. Error bars represent mean ± S.E.M., *n *=* *4.

The results in Figure 1 indicate that frataxin reach mitochondria. To ensure that TAT‐MTScs‐FXN is correctly processed by DRG neurons, cells were taken at day 5, lysed and protein samples analysed by Western blot to evaluate frataxin levels. The frataxin signal in TAT‐MTScs‐FXN‐treated DRG cultures is shown in Figure 2 where the full length, intermediate and mature forms can be detected. It is worth noting that in Fxn1 and Fxn2 cultures the levels of unprocessed construct were higher than those exhibited Scr cultures, suggesting a reduced ability to process frataxin. This could be a consequence of the fact that, as we previously reported 24, frataxin‐depleted neurons display decreased mitochondrial membrane potential (Δψ_m_) that could potentially affect TAT‐MTScs‐FXN penetration into the mitochondria. Indeed, Fxn1 and Fxn2 cultures displayed higher amounts of whole fusion protein and intermediate forms compared to control cultures (Scr) for each dose used (Fig. S2A and B). Interestingly, the level of mature FXN form in TAT‐MTScs‐FXN‐treated Fxn1 and Fxn2 cultures is not inferior to the level in Scr culture as one could expect (Fig. 2B).

### HSP60‐deficient processing is reversed by TAT‐MTScs‐FXN

**Figure 3:**
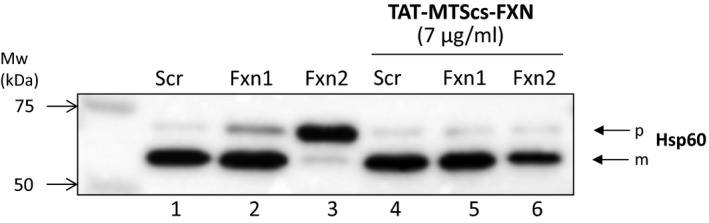
HSP60 processing in frataxin‐deficient neurons. Western blot detection of HSP60 precursor (p) and mature (m) forms in crude extracts from Scr, Fxn1 and Fxn2 cells after 5 days of culture without treatment (lanes 1–3) or treated with TAT‐MTScs‐frataxin at 7 μg/ml (lanes 4–6). Mw markers are indicated. Note that precursor form increases in untreated Fxn1 and Fxn2 cultures and the processing is restored by TAT‐MTScs‐FXN addition.

Impairment of the frataxin construct processing described above prompted us to analyse whether this was only affecting TAT‐MTScs‐FXN fusion protein or could also affect other mitochondrial proteins. For this reason, we analyse the HSP60, a mitochondrial molecular chaperone involved in protein folding, acting after the mitochondrial import machinery 28, 29. As HSP60 is targeted to mitochondria, it should also be processed to reach the mitochondrial matrix 30. Figure 3 (lanes 1–3) shows that in Fxn1 and Fxn2 cells, the precursor form (p) is increased with respect to control cultures (Scr), and accordingly, mature (m) form is decreased. As this could be a direct consequence of low frataxin levels displayed in Fxn1 an Fxn2 cultures, we tested the effect of adding TAT‐MTScs‐FXN to these cultures. As shown in Figure 3 (lanes 4–6), the levels of precursor and mature forms displayed in Fxn1 and Fxn2 are as those present in control Scr cultures, indicating that the mature frataxin form generated in Fxn1 and Fxn2 by TAT‐MTScs‐frataxin was sufficient to revert the anomalous processing of the molecular chaperone.

### Fodrin cleavage is reduced by TAT‐MTScs‐FXN

**Figure 4:**
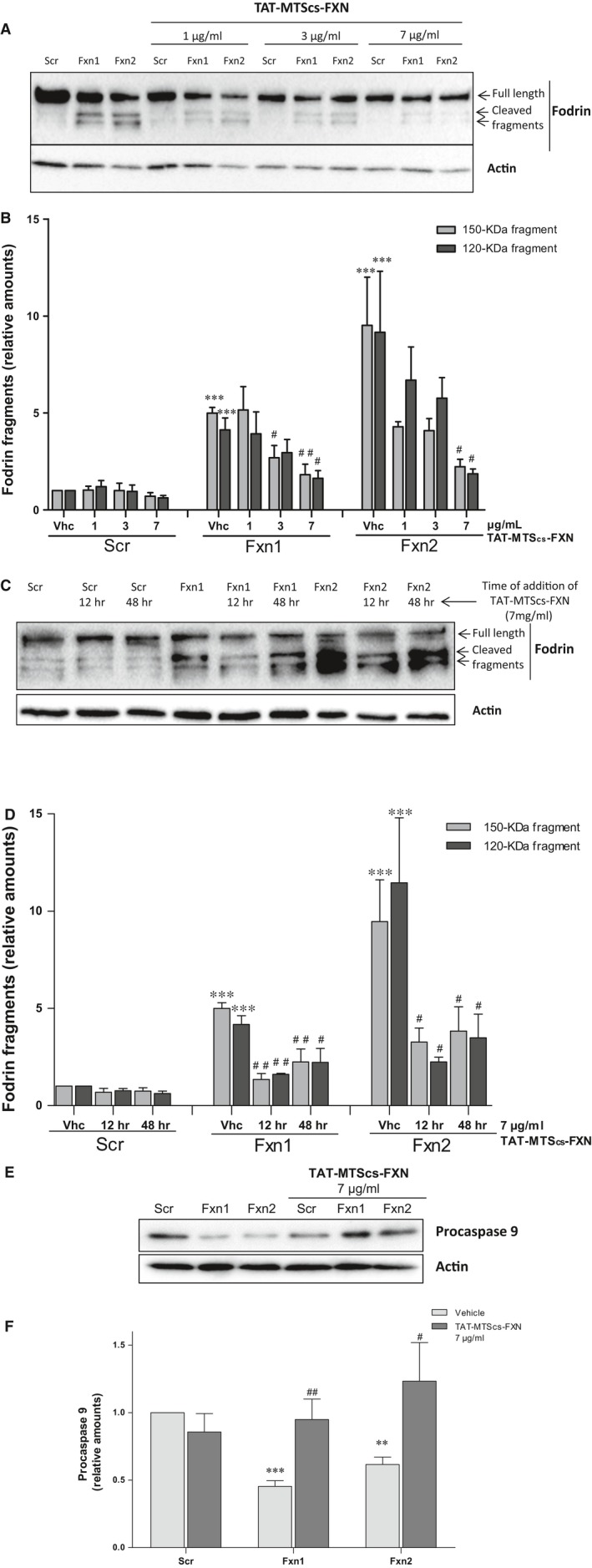
Fodrin cleavage is reduced by TAT‐MTScs‐FXN treatment (**A**) Western blotting of α‐Fodrin fragmentation. Crude extracts from Scr, Fxn1 and Fxn2 cells treated with increasing doses of 1, 3 and 7 μg/ml TAT‐MTScs‐FXN or vehicle solution 48 hrs after lentiviral transduction were checked for α‐Fodrin fragmentation (β‐actin was used for normalization). TAT‐MTScs‐FXN caused reduction in α‐fodrin cleavage as judged by the decrease of fodrin fragments (150 and 120 kD) in a dose‐dependent manner. (**B**) Histogram showing the decrease in relative amounts of fragmented α‐fodrin shown in A as function of the amount of TAT‐MTScs‐FXN added to cultures. (**C**) α‐fodrin cleavage after 5 days analysed in Scr, Fxn1 and Fxn2 cells treated with 7 μg/ml TAT‐MTScs‐FXN or vehicle solution 12 or 48 hrs after lentiviral transduction. The histogram in (**D**) represents the values of fodrin fragments shown in (**C**) to illustrate the differences of adding the compound at 12 or 48 hrs. Values are referred to untreated control (Scr) culture. In (**E**), procaspase 9 levels are shown in Scr, Fxn1, Fxn2 treated with TAT‐MTScs‐FXN and untreated. Note that the decreased amounts in untreated Fxn1 and Fxn2 are re‐established by TAT‐MTScs‐FXN addition. The histogram in (**F**) represents the values shown in (**E**). Asterisks (*) show significant values compared with control conditions (Scr); bars with **#** indicate significant differences with respect to the corresponding untreated control culture. Error bars represent mean ± S.E.M. In all figures, N is at least *n *=* *4.

The ability of TAT‐MTScs‐FXN to restore mitochondrial fitness to improve HSP60 processing leads us to analyse the reduction in apoptotic markers. In a previous study, we reported that apoptotic markers such as caspase 3 activation or α‐fodrin cleavage were clearly observed 24. In response to intracellular free Ca^2+^ increase, calpain and caspase proteases are activated, and among others, they cleave α‐fodrin, a cytoskeletal protein of 280 kD, rendering protease‐specific breakdown products (SBDPs) 31. Both proteases cleave the α‐fodrin to produce a non‐specific 150‐kD breakdown fragment (SBDP150), and then, a second cleavage results in a calpain or caspase 3‐specific product of 145 (SBDP145) or 120 kD (SBDP120), respectively. The presence of SBDP120 is considered a marker of apoptotic process 32. Based on these results, we evaluated the therapeutic effect of TAT‐MTScs‐FXN on frataxin‐deficient DRG neurons. Treating Scr and Fxn cultures with different doses of TAT‐MTScs‐FXN (1, 3 and 7 μg/ml) or vehicle solution at different time‐points (48 and 12 hrs after lentiviral transduction), we observed that TAT‐MTScs‐FXN concentrations reduced α‐fodrin cleavage in a dose‐dependent manner (Fig. 4A). A histogram showing such decrease in each culture is presented in Figure 4B. Also, addition of TAT‐MTScs‐FXN has a higher impact on reduction in fodrin cleavage when added shortly after lentivirus transduction. As shown in Figure 4C and D, addition of TAT‐MTScs‐FXN 7 μg/ml at 12 hrs after lentiviral transduction promotes optimal decrease in SBDPs as compared with results obtained by adding TAT‐MTScs‐FXN at 48 hrs. These results are also in agreement with those obtained by analysing caspase 9 activation, a marker of intrinsic or mitochondrial apoptotic pathway (for a review see 33). In Figure 4E and F, reduced levels of the precursor form are observed in Fxn1 and Fxn2 cells, indicative of cleavage (activation) of this caspase. When treated with TAT‐MTScs‐FXN, these cells display caspase 9 amounts similar to those observed in Scr cultures. These results show that apoptotic traits are reduced by TAT‐MTScs‐FXN and, consequently, degeneration of frataxin‐deficient DRG neurons should be prevented.

### Neurite degeneration and neuronal death are reduced by TAT‐MTScs‐FXN

**Figure 5:**
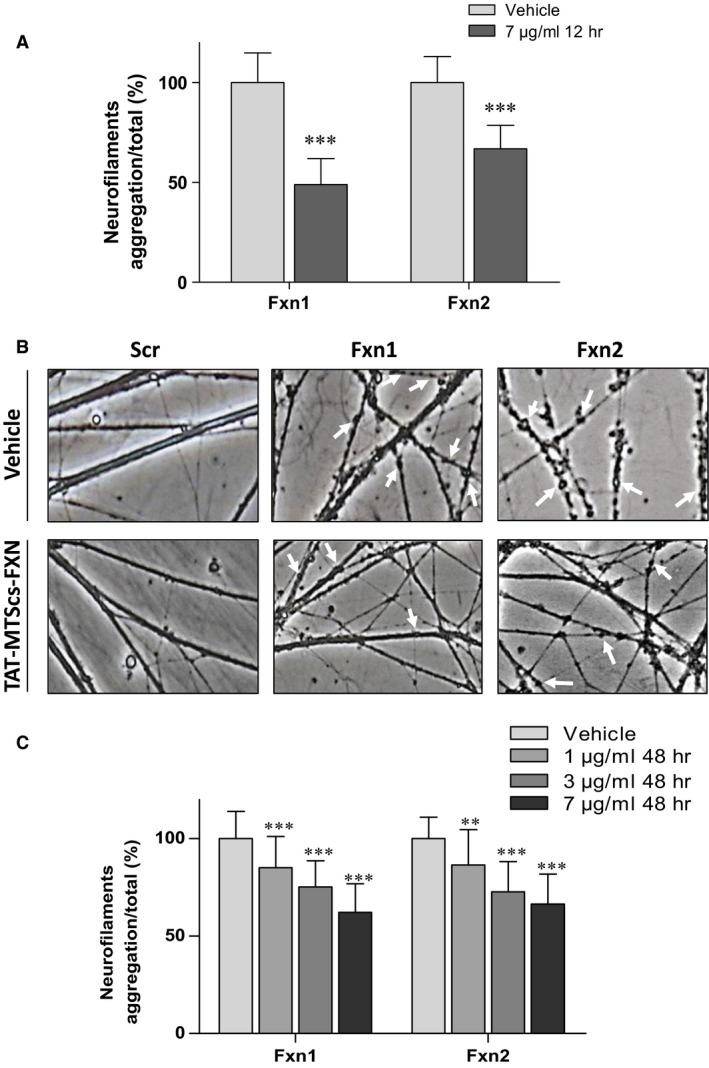
Neurite degeneration is reduced by TAT‐MTScs‐FXN treatment. (**A**) Histogram showing the significant decreased percentage of neurite degeneration (measured as described in Materials and methods) produced by TAT‐MTScs‐FXN treatment using 7 μg/ml after 12 hrs compared to untreated cells. Error bars represent mean ± S.D., *n *=* *4. (**B**) Representative images of 5‐day cultures of Scr, Fxn1 and Fxn2 treated with 7 μg/ml TAT‐MTScs‐FXN or vehicle solution 12 hrs after lentiviral transduction. Note the decrease in neurite aggregates (indicated by arrows) in treated cells (lower panels) compared with untreated cultures (upper panels). (**C**) Histogram showing the significant decreased percentage of neurite degeneration (measured as described in Materials and methods) produced by TAT‐MTScs‐FXN treatment (using 1 μg/ml, 3 μg/ml and 7 μg/ml) compared to untreated cells. Error bars represent mean ± S.D., *n *=* *4.

**Figure 6:**
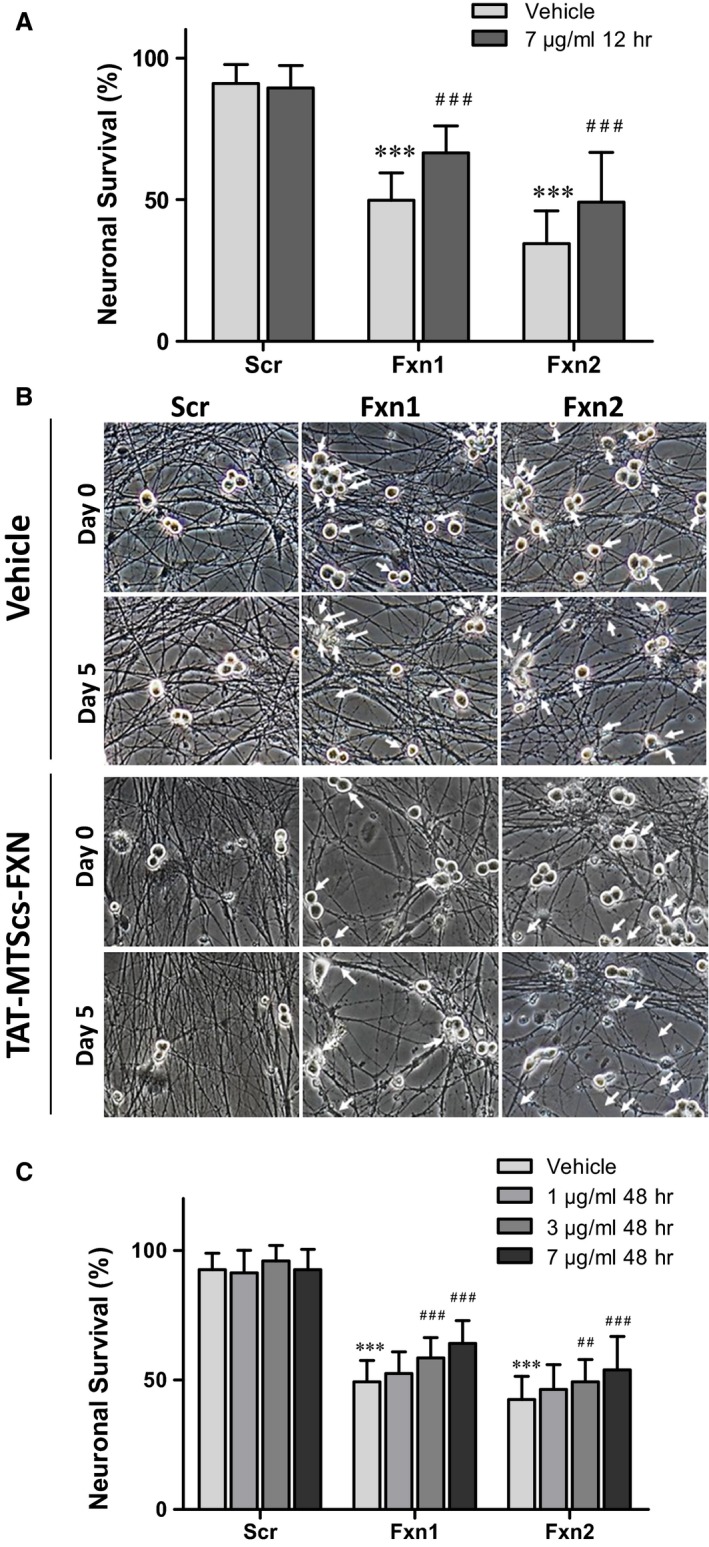
Cell survival is enhanced by TAT‐MTScs‐FXN treatment. (**A**) shows the percentage of surviving DRG neurons at day 5 of cultures treated with TAT‐MTScs‐FXN (7 μg/ml, 12 hrs post‐lentivirus transduction) compared to untreated cells. Note the significant survival increase by TAT‐MTScs‐FXN supplementation in both Fxn1 and Fxn2 cultures. Error bars represent mean ± S.D., *n *=* *4, and a range of 956–1184 neurons was scored for Scr conditions and 1397–1733 for each Fxn1 and Fxn2 conditions. (**B**) Representative images of neuronal survival with or without treatment (corresponding to 1/4^th^ of full‐size field). Images were taken at the same fields of cultures at day 0 and day 5. Arrows indicate the neuronal bodies present at day 0 and absent at day 5. In (**C**), the effect on survival of DRG‐deficient neurons when treated with TAT‐MTScs‐FXN added at 48 hrs after lentivirus transduction is shown. Significance is marked as (*) when referred to no treatment in Scr cultures and (#) when comparing 1, 3 and 7 μg/ml treatments. Error bars represent mean ± S.D., *n *=* *4, a range of 436–1233 neurons were scored for Scr condition and 867–1832 for each Fxn1 and Fxn2 conditions.

Frataxin depletion in DRG neurons promotes apoptosis and neurite degeneration, indicated by loss of mitochondrial membrane potential and formation of neurofilament aggregates 24. To further evaluate the positive effects of TAT‐MTScs‐FXN on frataxin‐deficient DRG neurons, neurite degeneration was analysed in our cultures. As shown in Figure 5A, addition of TAT‐MTScs‐FXN (7 μg/ml, 12 hrs after lentiviral transduction) to Fxn1 and Fxn2 cultures reduced neurofilament aggregates in neurites by 50% in Fxn1 and 35% in Fxn2. Images are shown in Figure 5B where aggregates are marked with arrows. In Figure 5C, we show that neurite degeneration is reduced in a concentration‐dependent manner, with 1, 3 and 7 μg/ml treatment after 48 hrs of transduction. Control Scr cultures were not affected by TAT‐MTScs‐FXN treatment (Fig. 5B, panels on the left). Besides neurite degeneration, Fxn1 and Fxn2 cultures showed a drastic decreased survival in accordance with increased apoptosis 24. For this reason, we analysed the ability of TAT‐MTScs‐FXN to recover survival in frataxin‐deficient DRG neurons. As shown in Figure 6A and B, addition of TAT‐MTScs‐FXN to Fxn1 and Fxn2 cultures after 12 hrs of lentiviral transduction resulted in a significant decrease in the percentage of mortality. At day 5, these cultures showed an increased survival rate of 16.6% and 14.6% per cent, respectively. When the compound was added 48 hrs after lentivirus transduction (Fig. 6C), the compound was still able to rescue the cultures from cell death although, compared to values achieved with TAT‐MTScs‐FXN added at 12 hrs, survival increased to a lesser extent (14.9% and 11.4% for 7 μg/ml and 9.2% and 6.8% for 3 μg/ml, in Fxn1 and Fxn2, respectively). Treatment with 1 μg/ml did not show any significant effect on cell survival. From these results, it can be concluded that in cultured frataxin‐deficient DRG neurons, TAT‐MTScs‐FXN is able to reduce neurite degeneration and, consequently, increase survival.

### Organ penetration, mitochondrial localization of TAT‐MTScs‐Frataxin in FXN‐KIKO mouse model

**Figure 7:**
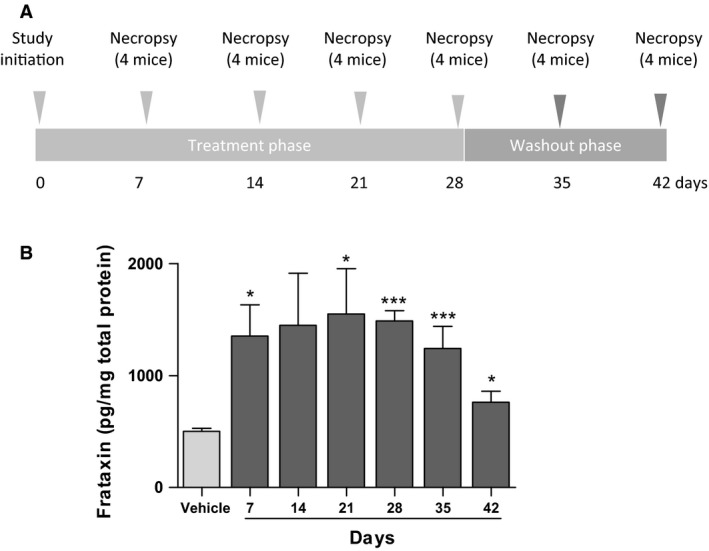
TAT‐MTScs‐FXN treatment FXN‐KO mice. (**A**) Study design: FA mice model 25 was treated with TAT‐MTScs‐FXN for 4 weeks followed by 2‐week washout. Mice were killed, muscles were harvested at the indicated time‐points (*n *=* *4 per time‐point), and frataxin amounts were evaluated in mitochondrial fractions as described in Materials and methods. (**B**) Frataxin amounts (expressed in pg/mg of total protein) present in muscle mitochondria of mice treated with 10 mg/kg with TAT‐MTScs‐FXN compared to untreated control (as indicated in the first column). Error bars represent mean ± S.E.M., *n *=* *4 per time‐point.

To test whether TAT‐MTScs‐FXN was also able to reach mitochondria *in vivo*, we used two mice models developed to study FA. For organ penetration and mitochondrial localization studies, mice model, described by Sarsero *et al*. 25, was IV injected with TAT‐MTScs‐FXN twice a week at 10 mg/kg dose, followed by 2 weeks of washout period (Fig. 7A). Mitochondrial fractions were obtained from muscles harvested at each time‐point. As can be seen (Fig. 7B), muscle Frataxin levels in mitochondria increased one week after study initiation, reaching highly significant level after three weeks of drug administration in comparison with untreated mice. The values show a significant increase of more than threefold compared to untreated mice that show values around 550 pg/mg. It is worth mentioning that the maximum values achieved of 1500 pg/mg of total protein at 21 days, represents around 15% of the normal values (*i.e*. concentration is typically around 10,300 pg/mg of total protein in muscles of healthy animals—data not shown). Interestingly, during the washout period, 2 weeks after ceasing administration, the frataxin levels were reduced but still remained significantly above (around twofold) compared to those of untreated mice. Besides to evaluate the increase of frataxin amounts in tissues other than muscle, brains from treated and control animals were used and shown in Figure S3.

### Mitochondrial functions restored with TAT‐MTScs‐Frataxin

**Figure 8:**
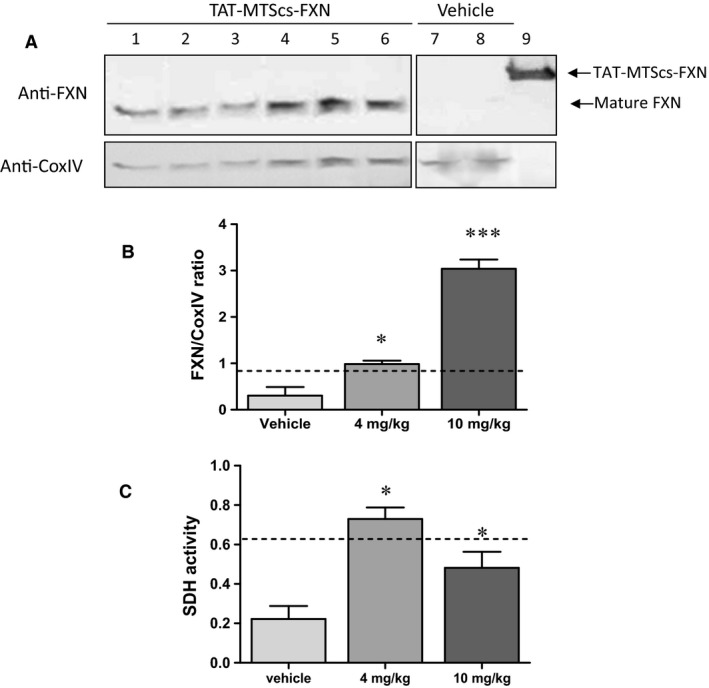
TAT‐MTScs‐FXN is processed and functional in mice muscle (**A**) A representative image of frataxin present in mitochondrial fractions obtained from mice treated for 3 weeks—twice a week—with 10 mg/kg TAT‐MTScs‐FXN (lanes 1–6) or vehicle (lanes 7–8 where frataxin is almost undetectable). The same protocol was executed by administrating 4 mg/kg (not shown). Each lane (1–6) represents individual mouse. Unprocessed external TAT‐MTScs‐FXN protein was run in parallel (lane 9). Lower panel shows the levels of COX‐IV that was used as internal mitochondrial marker. The frataxin levels normalized against COX‐IV are shown in (**B**). Dashed line indicates the frataxin levels of heterozygous, healthy mice. Bars represent mean ± S.E.M., *n *=* *4. In (**C**), the activity of mitochondrial complex II (succinate dehydrogenase) is shown. Values were obtained in samples of muscle mitochondrial fractions from mice treated with 4 and 10 mg/kg of TAT‐MTScs‐FXN and vehicle. Dashed line indicates the frataxin values for heterozygous, healthy mice. Results are presented as ΔOD at 0–10 min. per total protein (bars represent mean ± S.E.M., *n *=* *4, *P *<* *0.05,anova).

The KIKO‐FA model described above, provided that frataxin is reaching mitochondria, should display an improved phenotype. Hence, to examine frataxin function in the mitochondria, we used the MCK‐conditional mouse model 14 because striated muscle tissue is depleted of frataxin. As shown in Figure 8A, processed frataxin is clearly observed in muscle mitochondrial fractions obtained from mice treated with TAT‐MTScs‐FXN. For comparison, a mitochondrial protein, CoxIV, was used as internal standard. The presence of frataxin in muscle was dose‐dependent as, as shown in Figure 8B, animals treated with 10 mg/kg showed higher amounts (around threefold increase) compared to those treated with 4 mg/kg. To examine the ability of this frataxin to restore mitochondrial functions, the activity of succinate dehydrogenase (complex II) was tested. This is of special interest because deficiencies in succinate dehydrogenase have been associated with FA 10 and late‐onset neurodegeneration characterized by progressive optic atrophy, ataxia and myopathy 34. As shown in Fig. 8C, the enzymatic activity of SDH was increased, using either 4 mg/kg or 10 mg/kg, when compared to vehicle‐treated mice and restored the enzymatic activity values to those exhibited by heterozygous healthy mice (dashed line), thus indicating that the amount of frataxin in muscle is sufficient to partially restore mitochondrial functions.

### Survival of MCK mice treated with TAT‐MTScs‐FXN

**Figure 9:**
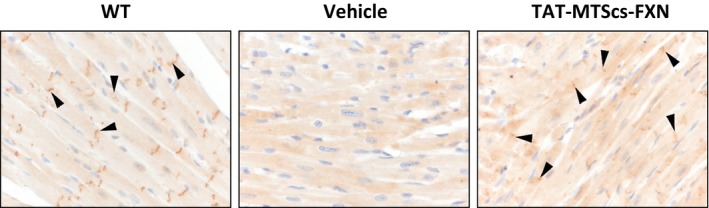
Frataxin detection in cardiac tissue of MCK mice model. Immunohistochemical staining of frataxin from WT, MCK vehicle‐treated and MCK TAT‐MTScs‐FXN‐treated mice are shown. Arrows indicate Frataxin accumulation near intercalated discs of cardiomyocytes for WT and MCK TAT‐MTScs‐FXN‐treated mice, but no frataxin were detected in MCK vehicle‐treated mice.

To test whether TAT‐MTScs‐FXN was able to rescue MCK phenotype, mouse survival was analysed as described in Materials and methods. Using intravenous route (IV) of injection, we did not observe a significant increased survival (Fig. S4). The reason for this could rely on the fact that when IV was used, amounts of frataxin in heart mitochondria of treated animals did not vary substantially when compared to controls (Fig. S5). MCK phenotype is mainly related to cardiac disfunctions. Firstly, we tested different ways of administration to deliver TAT‐MTScs‐FXN to heart tissue. Intraperitoneal injections resulted in an improvement compared to SC, IM and IV (Fig. S5). To test the presence of frataxin in cardiomyocytes, we performed immunohistochemistry on heart tissue treated with vehicle or TAT‐MTScs‐FXN. As shown in Figure 9, frataxin was undetectable in vehicle‐treated MCK mice while wild‐type mice and TAT‐MTScs‐FXN‐treated MCK mice show accumulation of frataxin near intercalated discs of cardiomyocytes. Indeed, mitochondria have been shown to be associated with intercalated discs in cardiomyocytes 35. Moreover, the disorganization of the muscle fibres of MCK mice, a key trait of the model 36, was partially restored with 10 mg/kg TAT‐MTScs‐FXN treatment.

**Figure 10:**
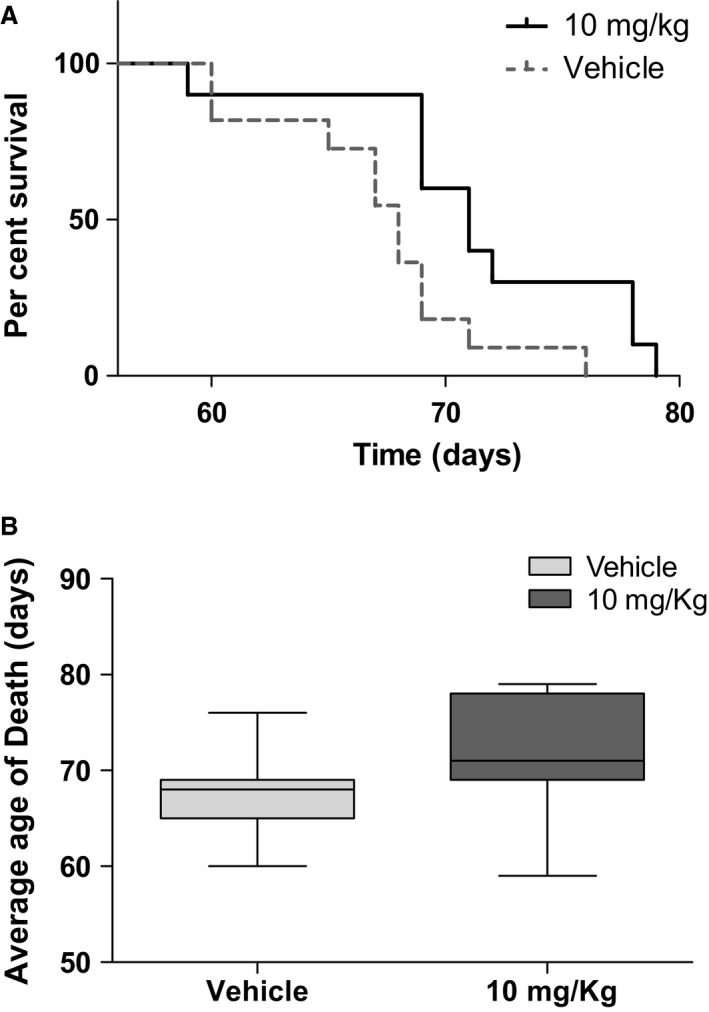
Survival of TAT‐MTScs‐Frataxin‐treated MCK mice. (**A**) Kaplan–Meier curve generated by GraphPad Prism showing the lifespan increase in MCK‐conditional mouse model treated with TAT‐MTScs‐FXN, *P *=* *0.0162 using log‐rank (Mantel–Cox) test. (**B**) Age of death, average was calculated using Gehan–Breslow–Wilcoxon test generated by GraphPad Prism, *P *=* *0.0251. Mice treated with 10 mg/kg of TAT‐MTScs‐FXN or vehicle (IP injection) were tested for survival (as described in Materials and methods). *N *=* *11 for each group.

Then, for survival assays, MCK mice were treated with IP injections of 4 mg/kg or 10 mg/kg. Although at 4 mg/kg they did not show significant differences with respect to vehicle (not shown), treatment with 10 mg/kg (Fig. 10A) shows an increase in total lifespan. The increased lifespan become evident in the interval from 65 to 75 days, where the maximum differences were observed. The mean survival was significantly higher for treated mice compared to control mice (67 *versus* 71 days, respectively). Moreover, in Figure 10B, age of death distribution is shown and the beneficial effects of the treatment are evident for the great majority of mice.

## Discussion

Frataxin depletion is the main cause of Friedreich ataxia. To date, there are no cures available and many efforts are currently made to develop a treatment for this disease (for an updated review of the different therapeutic approaches see *curefa.org/pipeline*). One of the strategies consists in replacing frataxin by an exogenous source as TAT‐based carrier molecule: TAT‐FXN. This technology allows cell penetration and mitochondria localization of exogenous FXN in FA mice model *in vivo* and in cell derived from FA patients 13, 15. Recently, TAT‐MTScs‐FXN was shown to be more efficient than TAT‐MTSfxn‐FXN in mitochondria localization 23. Pioneering studies conducted at Payne's laboratory showed that TAT‐frataxin fusion protein can deliver frataxin to mitochondria and increase lifespan of animal models of the disease by 53% 11. In this study, we used TAT‐FXN‐based construct in a neuronal model of the disease and show that TAT‐MTScs‐FXN was able to penetrate frataxin‐deficient sensory neurons and display a mitochondrial staining pattern. The amount of mature frataxin in TAT‐MTScs‐FXN‐treated Fxn1 and Fxn2 neurons was greatly increased compared to vehicle. Using mouse models, we also showed that mitochondria isolated from treated mice display detectable amounts of the protein. The use of frataxin‐deficient neuronal and muscle tissues enabled us to examine whether alterations in TAT‐MTScs‐FXN processing occur. In DRG neurons, we observed an alteration in TAT‐MTScs‐FXN processing. In Fxn1‐ and Fxn2‐treated neurons, we observed an accumulation of unprocessed TAT‐MTScs‐FXN, and intermediate forms, compared to Scr neurons treated with the same amount of TAT‐MTScs‐FXN but not mature form. This apparent contradiction can be explained by the results reported by Nabhan *et al*. 2015. The study demonstrated that CCCP treatment, which reduce Δψ_m,_ of 293T cells, increased precursor form but not mature form which remains unaltered 37). Our findings would be in agreement with these results. In muscle samples obtained from mice models, we did not observe any accumulation of TAT‐MTScs‐FXN, suggesting that it is mostly processed. One can hypothesize that impairment in protein processing is due to altered mitochondrial functions induced by frataxin deficiency, including the decreases in mitochondrial membrane potential (Δψ_m_) and considering that mitochondrial alterations are probably stronger in frataxin‐deficient neurons than in frataxin‐deficient muscle cell. As Δψ_m_ is a critical hub for the efficiency of mitochondrial protein import 17 and matrix protein translocation additionally requires ATP for mtHSP70 activity 18, major TAT‐MTScs‐FXN accumulation in frataxin‐depleted DRG neurons than in Scr neurons could be explained by the shift of mitochondrial energetic state and the quality of mitochondria, as protein import activity can act for sensing mitochondrial fitness 38. For this reason, we explore whether it was a condition specifically related to the nature of the construct or other mitochondrial proteins can suffer such impairment. For this reason, we selected HSP60, a molecular chaperone that must be imported and processed to reach the mitochondrial matrix. As shown in Figure 3, HSP60 processing is clearly impaired in Fxn1 and more clearly in Fxn2. The difference between both cultures can be attributable to lower levels of frataxin in Fxn2 cultures (as shown in Fig. S1). HSP60 impaired processing was not reported yet in frataxin‐deficient cells, and it could be relevant for the disease. In this context, it is worth mentioning that, beyond its function assisting polypeptides to fold properly once imported into the mitochondria, defects affecting its molecular chaperone activity have been related to neurodegenerative diseases such as spastic paraplegia [39, 40, 41] or the MitCHAP 60 disease [42, 43]. The impact that such deficiency could have in frataxin‐depleted cells remains to be explored.

Concerning other neurodegenerative markers, we previously described 24 that frataxin‐deficient cells DRG neurons showed decreased Δψ_m_ and apoptotic traits such as increased neurite degeneration and α‐fodrin cleavage. The fragmentation of this structural protein has been observed in normal ageing 44 and neurodegenerative diseases such as Alzheimer disease 45. The results presented here show that TAT‐MTScs‐FXN was able to reduce α‐fodrin cleavage, neurite degeneration and cell death in a dose‐dependent manner. Also, when administered at 12 hrs after transduction, α‐fodrin cleavage is more efficiently reduced than when added at 48 hrs (Fig. 4C and D). Rescuing from apoptotic process is reasonably more difficult at 48 hrs than after 12 hrs. Indeed, α‐fodrin cleavage is produced by caspase 3 and calpain proteases, which become activated by altered calcium homoeostasis through activation of Bax, a pro‐apoptotic protein 46, 47. Fodrin cleavage summarizes the activation of pro‐apoptotic pathway involving proteins such as P‐CREB, BAX, and others 48 with the participation of altered levels of calcium. Reduction in this fragmentation implies a reduction in pro‐apoptotic factors and restoring calcium homoeostasis. Then, it is reasonable that reduction in α‐fodrin cleavage—as shown in Figure 4A and B—and increase in procaspase 9 (Fig. 4E and F) are both due to restoring frataxin levels. These results agree with decreased neurite degeneration (Fig. 5) and with the increased survival observed by supplementing the cultures with TAT‐MTScs‐FXN at several doses (Fig. 6).

Conditional mice models of the disease used in this study showed alterations in both aconitase activity and mitochondrial electron transport chain 14, thus compromising mitochondrial energy production. We showed here that mice injected with TAT‐MTScs‐FXN reaches the muscle mitochondria achieving 15% of the values displayed by healthy animals. Although the increase in these values is moderate, they showed to be able to restore succinate dehydrogenase activity (Fig. 8C). Concerning animal survival, although the differences in mean survival treated with 4 mg/kg were not significant, treatment with 10 mg/kg proved to be partially efficient as the mean survival showed a slight but significant increase, mainly in the interval of 60–70 days. Moreover, the age of death was significantly increased with 10 mg/kg treatment.

FA is characterized by gradual neurodegeneration that precedes fatal cardiac condition. Unfortunately, there is no suitable neuronal model that mimics neurodegeneration and allows screening for drugs efficiency. In this paper, we show the ability of TAT‐MTScs‐FXN to protect neurodegeneration of primary neurons that are frataxin‐depleted. These “*in vitro*” results combined with the data provided by tests performed “*in vivo*” using mice models of Friedreich ataxia suggest that TAT‐MTScs‐frataxin can be considered a candidate for the disease therapy.

## Conflict of interest

The authors confirm that there are no conflict of interests.

## Supporting information


**Figure S1** Frataxin levels in Scr, Fxn1 and Fxn2 after lentivirus transduction. Levels of frataxin were analysed by Western blotting in control (Scr) and frataxin‐depleted neurons (Fxn1 and Fxn2). Actin was used as a control of protein loading.
**Figure S2** Relative amounts of full length, processed (intermediate and mature) TAT‐MTScs‐frataxin forms. (A) The amounts of the frataxin forms were evaluated in Scr, Fxn1 and Fxn2 cultures after addition of TAT‐MTScs‐frataxin at 1, 3 or 7 μg/ml (as indicated) to each culture, 48 hrs after lentivirus transduction. Error bars represent mean ± S.E.M., *n *=* *4 (B) Endogenous frataxin levels compared to treated cells. Cells from Scr, Fxn1 and Fxn2 untreated and treated with 1 and 7 μg/ml TAT‐MTScs‐FXN 48 hrs after lentiviral transduction were lysed after 5 days of culture and blotted to detect frataxin levels (β‐actin was used for normalization). Note the increase in mature frataxin levels in treated cells compared to untreated cells.

**Figure S3** Increased frataxin in brain samples (Sarsero model). Brain tissue samples were assayed for frataxin amounts using ELISA test. Treatment was initiated at 6–8 weeks of age. Mice were intravenously injected twice a week with vehicle or TAT‐MTScs‐frataxin at 4 mg/kg. Values are expressed as pg/mg of whole tissue extract. *N *=* *3 for each condition.

**Figure S4** Per cent of survival by administrating TAT‐MTScs‐frataxin intravenously. Survival of mice treated with vehicle or with TAT‐MTScs‐frataxin at 10 mg/Kg was followed (see Materials and methods). For controls, 5 animals were used and 10 animals for treatment. Note that no significant differences were observed in neither in mean nor in total lifespan. *N *=* *5 for vehicle and *N *=* *10 for TAT‐MTScs‐FXN.
**Figure S5** Frataxin amounts in heart mitochondria of MCK mice as function of injection route. Hearts from MCK mice were assayed for frataxin amounts. Note that intraperitoneally (IP) Injected mice showed the higher amounts of frataxin compared to subcutaneously (SC), intramuscular (IM) or intravenous (IV). Values were expressed as picograms/total mitochondrial protein. *N *=* *3 for each condition.

## References

[1] Jayadev S , Bird TD . Hereditary ataxias: overview. Genet Med. 2013; 15: 673–83. 23538602 10.1038/gim.2013.28

[2] Koeppen AH . Friedreich's ataxia: pathology, pathogenesis, and molecular genetics. J Neurol Sci. 2011; 303: 1–12. 21315377 10.1016/j.jns.2011.01.010 PMC3062632

[3] Campuzano V , Montermini L , Molto MD , *et al* Friedreich's ataxia: autosomal recessive disease caused by an intronic GAA triplet repeat expansion. Science. 1996; 271: 1423–7. 8596916 10.1126/science.271.5254.1423

[4] Campuzano V , Montermini L , Lutz Y , *et al* Frataxin is reduced in Friedreich ataxia patients and is associated with mitochondrial membranes. Hum Mol Genet. 1997; 6: 1771–80. 9302253 10.1093/hmg/6.11.1771

[5] Gibson TJ , Koonin EV , Musco G , *et al* Friedreich's ataxia protein: phylogenetic evidence for mitochondrial dysfunction. Trends Neurosci. 1996; 19: 465–8. 8931268 10.1016/S0166-2236(96)20054-2

[6] Zhang Y , Lyver ER , Knight SAB , *et al* Mrs3p, Mrs4p, and frataxin provide iron for Fe‐S cluster synthesis in mitochondria. J Biol Chem. 2006; 281: 22493–502. 16769722 10.1074/jbc.M604246200

[7] Wang T , Craig EA . Binding of yeast frataxin to the scaffold for Fe‐S cluster biogenesis, Isu. J Biol Chem. 2008; 283: 12674–9. 18319250 10.1074/jbc.M800399200 PMC2335353

[8] Robbins AH , Stout CD . Iron‐Sulfur cluster in aconsitase. J Biol Chem. 1985; 260: 2328–33. 3972791

[9] Albracht S.P.J . The prosthetic groups in succinate dehydrogenase. Number and stoichiometry. Biochim Biophys Acta. 1980; 612: 11–28. 6244847 10.1016/0005-2744(80)90274-0

[10] Rötig A , de Lonlay P , Chretien D , *et al* Aconitase and mitochondrial iron–sulphur protein deficiency in Friedreich ataxia. Nat Genet. 1997; 17: 215–7. 9326946 10.1038/ng1097-215

[11] Moreno‐Cermeno A , Alsina D , Cabiscol E , *et al* Metabolic remodeling in frataxin‐deficient yeast is mediated by Cth2 and Adr1. Biochim Biophys Acta. 2013; 1833: 3326–37. 24100161 10.1016/j.bbamcr.2013.09.019

[12] Tamarit J , Obis È , Ros J . Oxidative stress and altered lipid metabolism in Friedreich ataxia. Free Radic Biol Med. 2016; 100: 138–46. 27296838 10.1016/j.freeradbiomed.2016.06.007

[13] Vyas PM , Tomamichel WJ , Pride PM , *et al* A TAT‐Frataxin fusion protein increases lifespan and cardiac function in a conditional Friedreich's ataxia mouse model. Hum Mol Genet. 2012; 21: 1230–47. 22113996 10.1093/hmg/ddr554 PMC3284115

[14] Puccio H , Simon D , Cossee M , *et al* Mouse models for Friedreich ataxia exhibit cardiomyopathy, sensory nerve defect and Fe‐S enzyme deficiency followed by intramitochondrial iron deposits. Nat Genet. 2001; 27: 181–6. 11175786 10.1038/84818

[15] Kim MJ , Kim DW , Jeong HJ , *et al* Tat‐Frataxin protects dopaminergic neuronal cells against MPTP‐induced toxicity in a mouse model of Parkinson's disease. Biochimie. 2012; 94: 2448–56. 22809528 10.1016/j.biochi.2012.07.005

[16] von Heijne G . Mitochondrial targeting sequences may form amphiphilic helices. EMBO J. 1986; 5: 1335–42. 3015599 10.1002/j.1460-2075.1986.tb04364.x PMC1166945

[17] Martin J , Mahlke K , Pfanners N . Role of an energized inner membrane in mitochondrial protein import. Δψ drives the movement of presequences. J Biol Chem. 1991; 266: 18051–7. 1833391

[18] Matouschek A , Pfanner N , Voos W , *et al* Protein unfolding by mitochondria. The Hsp70 import motor. EMBO Rep. 2000; 1: 404–10. 11258479 10.1093/embo-reports/kvd093 PMC1083766

[19] Sinha D , Joshi N , Chittoor B , *et al* Role of magmas in protein transport and human mitochondria biogenesis. Hum Mol Genet. 2010; 19: 1248–62. 20053669 10.1093/hmg/ddq002 PMC2838536

[20] Frazier AE , Dudek J , Guiard B , *et al* Pam16 has an essential role in the mitochondrial protein import motor. Nat Struct Mol Biol. 2004; 11: 226–33. 14981507 10.1038/nsmb735

[21] Cavadini P , Adamec J , Taroni F , *et al* Two‐step processing of human frataxin by mitochondrial processing peptidase precursor and intermediate forms are cleaved at different rates. J Biol Chem. 2000; 275: 41469–75. 11020385 10.1074/jbc.M006539200

[22] Schmucker S , Argentini M , Carelle‐Calmels N , *et al* The in vivo mitochondrial two‐step maturation of human frataxin. Hum Mol Genet. 2008; 17: 3521–31. 18725397 10.1093/hmg/ddn244

[23] Marcus D , Lichtenstein M , Cohen N , *et al* Heterologous mitochondrial targeting sequences can deliver functional proteins into mitochondria. Int J Biochem Cell Biol. 2016; 81: 48–56. 27771440 10.1016/j.biocel.2016.10.013

[24] Mincheva‐Tasheva S , Obis E , Tamarit J , *et al* Apoptotic cell death and altered calcium homeostasis caused by frataxin depletion in dorsal root ganglia neurons can be prevented by BH4 domain of Bcl‐xL protein. Hum Mol Genet. 2014; 23: 1829–41. 24242291 10.1093/hmg/ddt576

[25] Sarsero JP , Li L , Holloway TP , *et al* Human BAC‐mediated rescue of the Friedreich ataxia knockout mutation in transgenic mice. Mamm Genome. 2004; 15: 370–82. 15170226 10.1007/s00335-004-3019-3

[26] Perdomini M , Belbellaa B , Monassier L , *et al* Prevention and reversal of severe mitochondrial cardiomyopathy by gene therapy in a mouse model of Friedreich's ataxia. Nat Med. 2014; 20: 542–7. 24705334 10.1038/nm.3510

[27] Wadia JS , Stan RV , Dowdy SF . Transducible TAT‐HA fusogenic peptide enhances escape of TAT‐fusion proteins after lipid raft macropinocytosis. Nat Med. 2004; 10: 310–5. 14770178 10.1038/nm996

[28] Viitanen PV , Lorimer GH , Seetharam R , *et al* Mammalian mitochondrial chaperonin 60 functions as a single toroidal ring. J Biol Chem. 1992; 267: 695–8. 1346131

[29] Sigler PB , Xu Z , Rye HS , *et al* Structure and function in groel‐mediated protein folding. Annu Rev Biochem. 1998; 67: 581–608. 9759498 10.1146/annurev.biochem.67.1.581

[30] Peralta D , Lithgow T , Hoogenraad NJ , *et al* Prechaperonin 60 and preornithine transcarbamylase share components of the import apparatus but have distinct maturation pathways in rat liver mitochondria. Eur J Biochem. 1993; 211: 881–9. 8094670 10.1111/j.1432-1033.1993.tb17621.x

[31] Nath R , Raser KJ , Stafford D , *et al* Non‐erythroid α‐spectrin breakdown by calpain and interleukin 1β‐converting‐enzyme‐like protease(s) in apoptotic cells : contributory roles of both protease families in neuronal apoptosis. Biochem J. 1996; 319: 683–90. 8920967 10.1042/bj3190683 PMC1217843

[32] Pike BR , Flint J , Dave JR , *et al* Accumulation of calpain and caspase‐3 proteolytic fragments of brain‐derived a‐II‐spectrin in cerebral spinal fluid after middle cerebral artery occlusion in rats. J Cereb Blood Flow Metab. 2004; 24: 98–106. 14688621 10.1097/01.WCB.0000098520.11962.37

[33] Li P , Zhou L , Zhao T , *et al* Caspase‐9: structure, mechanisms and clinical application. Oncotarget. 2015; 8: 23996–4008. 10.18632/oncotarget.15098 PMC5410359 28177918

[34] Hoekstra AS , Bayley J‐P . The role of complex II in disease. Biochim Biophys Acta ‐ Bioenerg. 2013; 1827: 543–51. 10.1016/j.bbabio.2012.11.005 23174333

[35] Forbes MS , Sperelakis N . Association between and gap junctions in mammalian. Tissue Cell. 1982; 14: 25–37. 7089964 10.1016/0040-8166(82)90004-0

[36] Koeppen AH , Becker AB , Feustel PJ , *et al* The significance of intercalated discs in the pathogenesis of Friedreich cardiomyopathy. J Neurol Sci. 2016; 367: 171–176. 27423584 10.1016/j.jns.2016.06.006

[37] Nabhan JF , Gooch RL , Piatnitski Chekler EL , *et al* Perturbation of cellular proteostasis networks identifies pathways that modulate precursor and intermediate but not mature levels of frataxin. Sci Rep. 2015; 5: 18251. 26671574 10.1038/srep18251 PMC4680912

[38] Harbauer AB , Zahedi RP , Sickmann A , *et al* The protein import machinery of mitochondria ‐ a regulatory hub in metabolism, stress, and disease. Cell Metab. 2014; 19: 357–72. 24561263 10.1016/j.cmet.2014.01.010

[39] Hansen JJ , Dürr A , Cournu‐Rebeix I , *et al* Hereditary spastic paraplegia SPG13 is associated with a mutation in the gene encoding the mitochondrial chaperonin Hsp60. Am J Hum Genet. 2002; 70: 1328–32. 11898127 10.1086/339935 PMC447607

[40] Bross P , Naundrup S , Hansen J , *et al* The Hsp60‐(p. V98I) mutation associated with hereditary spastic paraplegia SPG13 compromises chaperonin function both in vitro and in vivo. J Biol Chem. 2008; 283: 15694–700. 18400758 10.1074/jbc.M800548200 PMC3259655

[41] Magnoni R , Palmfeldt J , Christensen JH , *et al* Late onset motoneuron disorder caused by mitochondrial Hsp60 chaperone deficiency in mice. Neurobiol Dis. 2013; 54: 12–23. 23466696 10.1016/j.nbd.2013.02.012

[42] Parnas A , Nadler M , Nisemblat S , *et al* The MitCHAP‐60 disease is due to entropic destabilization of the human mitochondrial Hsp60 oligomer. J Biol Chem. 2009; 284: 28198–203. 19706612 10.1074/jbc.M109.031997 PMC2788871

[43] Bross P , Fernandez‐Guerra P . Disease‐associated mutations in the HSPD1 gene encoding the large subunit of the mitochondrial HSP60/HSP10 chaperonin complex. Front Mol Biosci. 2016; 3: 49. 10.3389/fmolb.2016.00049 PMC5006179 27630992

[44] Bahr BA , Vanderklish PW , Ha LT , *et al* Spectrin breakdown products increase with age in telencephalon of mouse brain. Neurosci Lett. 1991; 131: 237–40. 1762696 10.1016/0304-3940(91)90622-z

[45] Ayala‐Grosso C , Tam J , Roy S , *et al* Caspase‐3 cleaved spectrin colocalizes with neurofilament‐immunoreactive neurons in Alzheimer's disease. Neuroscience. 2006; 141: 863–74. 16750894 10.1016/j.neuroscience.2006.04.041

[46] Suwanjang W , Phansuwan‐Pujito P , Govitrapong P , *et al* Calpastatin reduces calpain and caspase activation in methamphetamine‐induced toxicity in human neuroblastoma SH‐SY5Y cultured cells. Neurosci Lett. 2012; 526: 49–53. 22897874 10.1016/j.neulet.2012.07.066

[47] Kuwako K , Nishimura I , Uetsuki T , *et al* Activation of calpain in cultured neurons overexpressing Alzheimer amyloid precursor protein. Mol Brain Res. 2002; 107: 166–75. 12425945 10.1016/s0169-328x(02)00489-8

[48] Thippeswamy T , Morris R . The roles of nitric oxide in dorsal root ganglion neurons. Ann N Y Acad Sci. 2002; 962: 103–110. 12076967 10.1111/j.1749-6632.2002.tb04060.x

