# Supplementary figures and images for: Frataxin‐deficient neurons and mice models of Friedreich ataxia are improved by TAT‐MTScs‐FXN treatment
Source: J Cell Mol Med. 2017 Oct 5;22(2):834–48. doi: 10.1111/jcmm.13365 (PMC5783845; doi:10.1111/jcmm.13365)

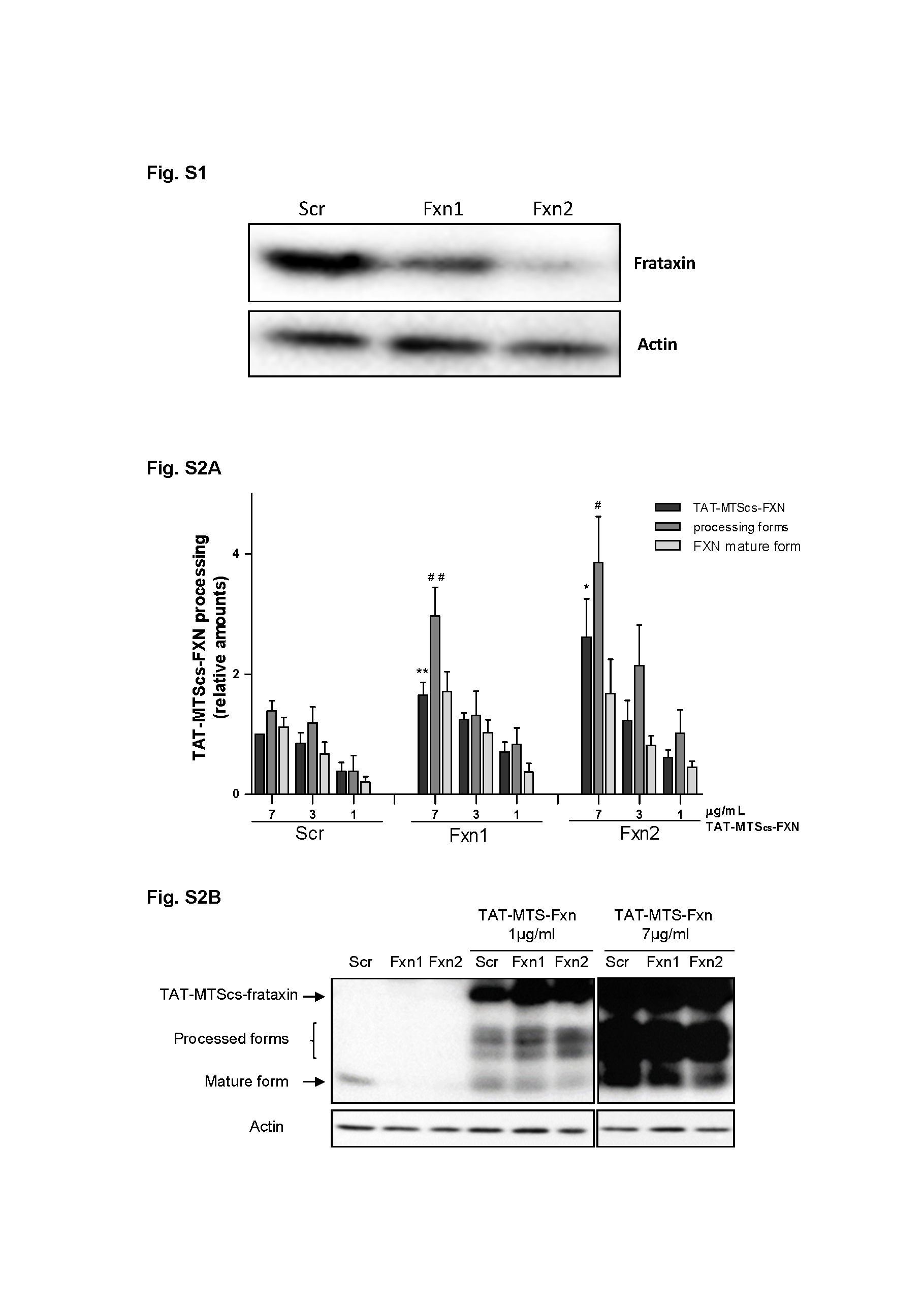

Supplement: Supplementary file 1 — Figure S1 Frataxin levels in Scr, Fxn1 and Fxn2 after lentivirus transduction. Levels of frataxin were analysed by Western blotting in control (Scr) and frataxin‐depleted neurons (Fxn1 and Fxn2). Actin was used as a control of protein loading. Figure S2 Relative amounts of full length, processed (intermediate and mature) TAT‐MTScs‐frataxin forms. (A) The amounts of the frataxin forms were evaluated in Scr, Fxn1 and Fxn2 cultures after addition of TAT‐MTScs‐frataxin at 1, 3 or 7 μg/ml (as indicated) to each culture, 48 hrs after lentivirus transduction. Error bars represent mean ± S.E.M., n = 4 (B) Endogenous frataxin levels compared to treated cells. Cells from Scr, Fxn1 and Fxn2 untreated and treated with 1 and 7 μg/ml TAT‐MTScs‐FXN 48 hrs after lentiviral transduction were lysed after 5 days of culture and blotted to detect frataxin levels (β‐actin was used for normalization). Note the increase in mature frataxin levels in treated cells compared to untreated cells. [file JCMM-22-834-s001.tiff]

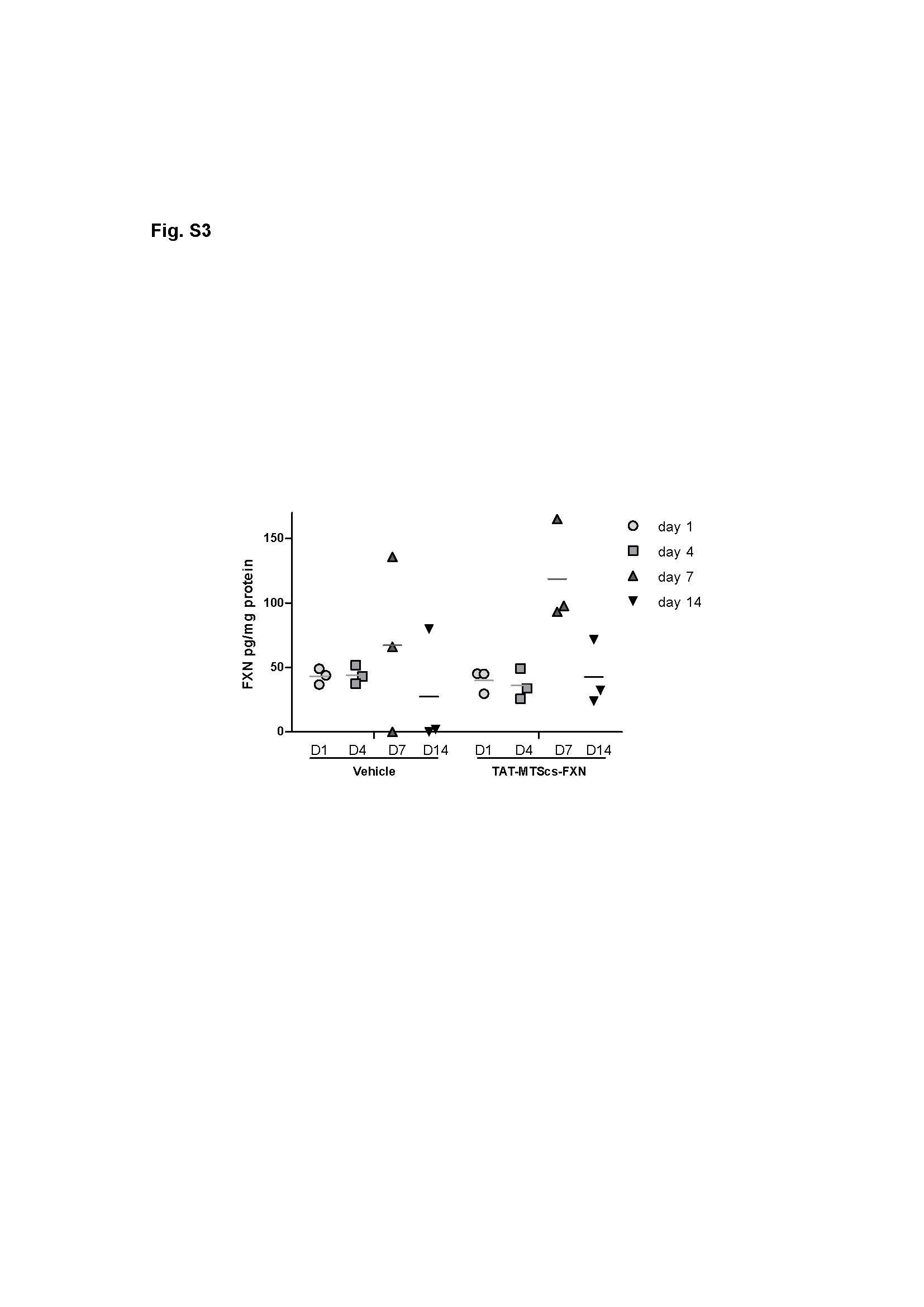

Supplement: Supplementary file 2 — Figure S3 Increased frataxin in brain samples (Sarsero model). Brain tissue samples were assayed for frataxin amounts using ELISA test. Treatment was initiated at 6–8 weeks of age. Mice were intravenously injected twice a week with vehicle or TAT‐MTScs‐frataxin at 4 mg/kg. Values are expressed as pg/mg of whole tissue extract. N = 3 for each condition. [file JCMM-22-834-s002.tif]

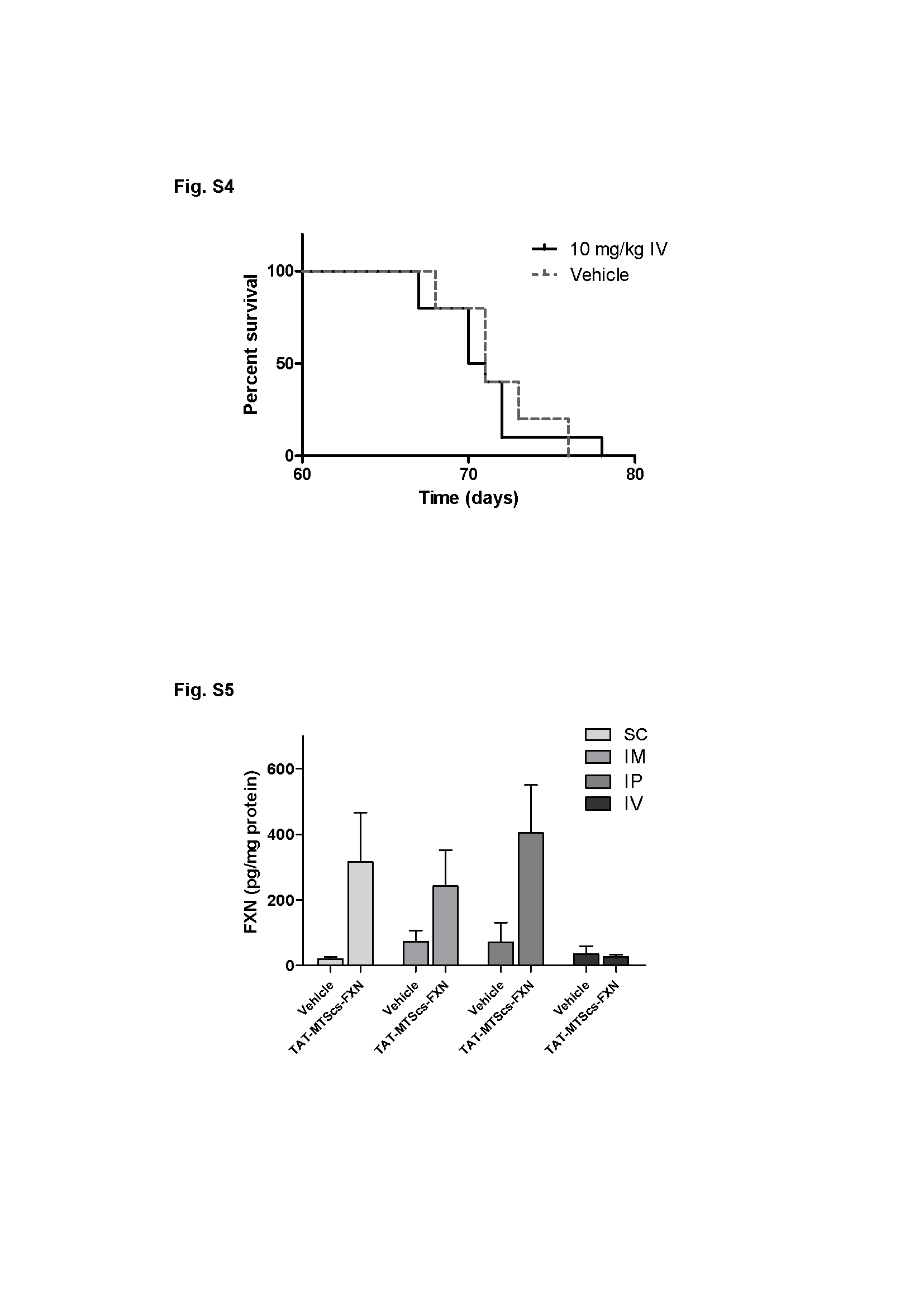

Supplement: Supplementary file 3 — Figure S4 Per cent of survival by administrating TAT‐MTScs‐frataxin intravenously. Survival of mice treated with vehicle or with TAT‐MTScs‐frataxin at 10 mg/Kg was followed (see Materials and methods). For controls, 5 animals were used and 10 animals for treatment. Note that no significant differences were observed in neither in mean nor in total lifespan. N = 5 for vehicle and N = 10 for TAT‐MTScs‐FXN. Figure S5 Frataxin amounts in heart mitochondria of MCK mice as function of injection route. Hearts from MCK mice were assayed for frataxin amounts. Note that intraperitoneally (IP) Injected mice showed the higher amounts of frataxin compared to subcutaneously (SC), intramuscular (IM) or intravenous (IV). Values were expressed as picograms/total mitochondrial protein. N = 3 for each condition. [file JCMM-22-834-s003.tif]
